# Residual Stress Analysis Based on Acoustic and Optical Methods

**DOI:** 10.3390/ma9020112

**Published:** 2016-02-16

**Authors:** Sanichiro Yoshida, Tomohiro Sasaki, Masaru Usui, Shuichi Sakamoto, David Gurney, Ik-Keun Park

**Affiliations:** 1 Department of Chemistry and Physics, Southeastern Louisiana University, Hammond, LA 70402, USA; dgurney@selu.edu; 2 Department of Mechanical Engineering, Niigata University, Ikarashi Ninocho 8050, Nishi-ku, Niigata-shi, Niigata 950-2181, Japan; tomodx@eng.niigata-u.ac.jp (T.S.); masa_baske_usubou07@yahoo.co.jp (M.U.); sakamoto@eng.niigata-u.ac.jp (S.S.); 3 Department of Mechanical and Automotive Engineering, Seoul National University of Science and Technology, 232 Gongneung-ro, Nowon-gu, Seoul 139-743, Korea; ikpark@seoultech.ac.kr

**Keywords:** residual stress analysis, acoustoelasticity, electronic speckle-pattern interferometry, scanning acoustic microscopy

## Abstract

Co-application of acoustoelasticity and optical interferometry to residual stress analysis is discussed. The underlying idea is to combine the advantages of both methods. Acoustoelasticity is capable of evaluating a residual stress absolutely but it is a single point measurement. Optical interferometry is able to measure deformation yielding two-dimensional, full-field data, but it is not suitable for absolute evaluation of residual stresses. By theoretically relating the deformation data to residual stresses, and calibrating it with absolute residual stress evaluated at a reference point, it is possible to measure residual stresses quantitatively, nondestructively and two-dimensionally. The feasibility of the idea has been tested with a butt-jointed dissimilar plate specimen. A steel plate 18.5 mm wide, 50 mm long and 3.37 mm thick is braze-jointed to a cemented carbide plate of the same dimension along the 18.5 mm-side. Acoustoelasticity evaluates the elastic modulus at reference points via acoustic velocity measurement. A tensile load is applied to the specimen at a constant pulling rate in a stress range substantially lower than the yield stress. Optical interferometry measures the resulting acceleration field. Based on the theory of harmonic oscillation, the acceleration field is correlated to compressive and tensile residual stresses qualitatively. The acoustic and optical results show reasonable agreement in the compressive and tensile residual stresses, indicating the feasibility of the idea.

## 1. Introduction

Analysis of residual stresses is a long-standing and challenging problem in many fields of engineering [1,2]. Residual stresses are developed during most manufacturing processes such as grinding, cold rolling, welding and other types of joining processes. Being hidden in a material and independent of an external load, they are easily overlooked. The level of residual stresses, on the other hand, is often significantly high. It is not unusual that a residual stress is comparable to the yield stress, shortening the life of the mechanical system or leading to a total failure upon an external impact. Vehicles used in motorsports have a number of joints, and the behavior of residual stresses can lead to disastrous consequences. As an example, fracture of the steering arm spindle assembly at the weld joint upon a collision is a common cause of fatal accidents in motorcycle races. It is obvious that residual stresses in the weld lowers the fracture strength of the assembly. Proper analysis and detection of residual stresses is certainly important for safety.

The fundamental complexity of the problem lies in the fact that a residual stress is locked in the material. To evaluate a residual stress quantitatively, we need to break the locking mechanism, or at minimum, change the stress status by applying a force to the locking mechanism. Techniques classified as destructive methods remove part of the material with various techniques [3,4,5,6], and measure the relaxed strain. While these methods are reliable and often developed to the level of field uses, they are not suitable for applications in which material removal is not favorable. Analysis of residual stresses in welded works [7,8] is a good example. Residual stresses induced by welding are often found in the heat-affected zone, which is normally weaker than the weld zone or the other part of the work. Removal of material can further weaken the heat-affected zone, and can compromise the overall mechanical strength of the work. For these applications, a nondestructive technique would be preferred.

Techniques classified as nondestructive methods do not involve a process to break the locking mechanism. These techniques can be classified into two general categories; static methods and dynamic methods. Acoustic imaging microscopy [9,10] and diffractometry [11,12] fall into the category of static methods. Acoustic imaging microscopy detects abnormality in residually-stressed regions. It can visualize the abnormality as a full-field image but it cannot quantify the stress. Diffractometry evaluates atomic rearrangement due to residual stresses. It measures the inter-atomic distance based on the diffraction pattern generated via interaction between X-Ray, neutron, synchrotron or similar radiation and the crystal lattice of the specimen.

Diffractometry relies on an elastic modulus to evaluate the residual stress from the measured atomic rearrangement. However, this process is not straightforward for two reasons. First, it is likely that the residual stress is so high that the constitutive relation is not linear anymore. Under this condition, it is inappropriate to use the elastic modulus of the linear regime for the estimation of the residual stress from the measured strain (the atomic rearrangement). In fact, as will be discussed shortly, acoustoelasticity utilizes the nonlinearity in the constitutive relation to evaluate residual stresses. Assuming a nonlinear constitutive relation is legitimate for residual stress analysis. Second, often permanent (hence plastic) deformation of the surrounding material is the locking mechanism of a residual stress. In that case, it is inaccurate to use the elastic modulus to calculate a residual stress from measured strain. If the strain is partly due to plastic deformation, we would overestimate the residual stress (because the actual modulus of the region is less than the elastic modulus). That exact problem occurs in the destructive methods that use an elastic modulus to evaluate a residual stress from relaxed strain.

Techniques classified as dynamic methods apply a certain load to the residually-stressed region, and measure the response. Acoustoelasticity [13,14,15,16,17,18] and thermal methods [19,20] fall into this category. Acoustoelasticity applies a cyclic load at the acoustic frequency and thermal methods apply a thermal load by heating. It should be emphasized that the strain resulting from the applied load does not represent the residual stress unless the load completely relaxes the residual stress. If the load does not relax the residual stress, the strain is associated with the stress due to the applied load, not to the residual stress. Acoustoelasticity evaluates the deviation of the elastic modulus from the nominal value (the elastic modulus with no stress) through the measurement of acoustic velocity. Provided that the strain potential energy curve is known, the deviation of the elastic modulus reveals the atomic displacement from the equilibrium, hence the residual strain. Since the elastic modulus is known, it is possible to determine the residual stress absolutely from the strain data. Thermal methods relax the residual stress with the thermal load.

Acoustoelasticity is widely used and quite attractive. Since this technique evaluates the elastic modulus, it solves the above-mentioned issue of the uncertainty in the use of a proper elastic constant associated with the nonlinearity of the constitutive relation. The acoustic wave for the velocity measurement can be in the form of a bulk wave [13,14,15,16], a surface wave [17,18], or in other forms as long as the velocity is defined. Thus, the hardware setup is relatively easy to configure. However, this technique still has two drawbacks. First, it is essentially a point-wise measuring technique. This feature not only makes the measurement time-consuming but also inaccurate in situations where the residual stress varies rapidly as a function of the spatial coordinates. Our previous experiment [21] on butt-welding induced residual stresses has shown that the thermal load makes deformation near the weld-line oscillatory, indicating the possibility that the residual stress varies rapidly along the weld-line. Missing a point where the residual stress has a steep spatial gradient can result in incorrect interpretation of the measurement; therefore, a full-field method is preferred. Second, the above-mentioned issue of inaccuracy associated with the coexistence of plastic deformation in the residually-stressed region applies to acoustoelasticity.

Considering the above situations, we propose co-application of acoustoelasticity and optical interferometry. Here, acoustoelasticity is used to evaluate the elastic modulus at reference points, and optical interferometry is used to analyze the material’s dynamic response to an external load under the influence of residual stresses. It is relatively easy to configure the optical interferometer to generate a full-field, two dimensional map of displacement or its derivatives such as velocity and strain. By hypothesizing that the increases/decreases in the local elastic modulus due to a compressive/tensile residual stress are high enough to alter the dynamic behavior of the material, and that the resultant change in the dynamic behavior is detectable as a certain pattern of the velocity field measured with the optical interferometer, we can configure an algorithm to correlate the residual stress and its distribution to a two-dimensional, full-field map of the velocity field.

Furthermore, by developing an algorithm to differentiate elastic deformation from plastic deformation in a given point of the displacement field or their derivatives generated by the optical interferometer, it is possible to ascertain that the constitutive modulus revealed by acoustoelasticity at the reference point reflects pure elastic behavior, and thereby prevent the above-mentioned overestimation of the residual stress. If all these ideas are verified, we can design a system capable of converting the full-field residual stress distribution to a map of absolute residual stresses. The development of such an algorithm is certainly challenging, but our recent study [22] indicates some encouraging results.

In the study [22], we measured the strain field of a butt-welded, thin steel specimen using an optical interferometer, and were able to differentiate between elastically and plastically deformed regions based on a comprehensive theory of deformation [23]. The analysis showed clear consistence with stress behavior analyzed with conventional strain gauge measurement conducted independently on the same specimen.

The aim of this paper is to report on our recent experimental study on the above idea of co-applying acousto-optical methods to residual stress analysis. Using two acoustic techniques and an optical interferometric technique on a butt-brazed dissimilar metal plate (steel and cemented carbide) as a sample specimen, we were able to demonstrate the feasibility of the idea qualitatively. The three techniques used are (a) acoustoelasticity with the use of bulk acoustic longitudinal and shear waves (called the contact-acoustoelastic technique) [13,14,15,16,17,18]; (b) acoustoelasticity with the use of a surface acoustic wave (Scanning Acousto Microscopy, SAM) [24,25,26,27,28,29,30]; and (c) optical interferometry known as the Electronic Speckle-Pattern Interferometry (ESPI).

Measurements with contact-acoustoelastic technique indicate that in-plane compressive residual stresses are concentrated near the butt-joint line on the steel side. Measurements with SAM indicate that out-of-plane compressive residual stresses are concentrated within 20 μm of the surface of the same region. The cemented carbide side shows rather uniform tensile residual stresses in the out-of-plane direction, and compressive residual stresses in the in-plain directions.

The in-plane acceleration field derived from the velocity field generated with ESPI accounts for the elastic modulus being altered by the observed residual stresses in a consistent fashion with the acoustic measurement. This result qualitatively supports the above hypothesis that the dynamic behavior associated with the elastic modulus altered by the residual stress can be characterized by the optical method in the form of full-field information. The “Experimental” Section below describes the principle of operation along with experimental procedures. The “Results and Discussion” Section discusses the experimental results and assesses the proposed method. Although this paper reports experiments on the butt-brazed plate specimen as an example, the method is applicable to other cases in general provided that the same experimental procedures can be performed.

## 2. Experimental

### 2.1. Principle of Operation

The above three techniques utilize the advantages of the respective methods and compensate for their drawbacks. The contact-acoustoelastic technique is capable of determining residual strains quantitatively via measurement of acoustic velocity and its deviation from the nominal value (the acoustic velocity with no stress of the same material). With the single transducer configuration and a well-defined acoustic wave, it is relatively easy to identify the elastic modulus of the specimen. However, the technique does not have depth sensitivity as the measured acoustic velocity represents the change in the elastic modulus over the entire thickness of the specimen. Often, residual stresses in plate specimens are localized near the surface. SAM, on the other hand, is sensitive to the depth information [26]. The surface acoustic wave is formed with an evanescent acoustic wave that penetrates through the surface and decays exponentially with the distance from the surface. Since the penetration depth is inversely proportional to the acoustic frequency, use of various acoustic frequencies provides us with acoustic velocity information at different depths. However, the acoustic wave is not as well defined as the contact transducer case. It is a mixture of longitudinal and shear components of a wave, and the ratio of the components depends on the thickness and other structural parameters. This feature complicates the estimation of the elastic modulus from the measured acoustic velocity as compared with the contact-acoustoelastic technique. Both acoustic techniques are point-by-point methods.

The ESPI technique yields a full-field, two-dimensional map of displacement but it requires application of an external load. We applied a tensile load to the specimen at a constant pulling rate in a stress range substantially lower than the yield stress, and measured the resultant displacement using an ESPI setup sensitive to in-plane displacement. The type of the ESPI technique used in this study [31,32] is able to evaluate the displacements occurring in a short interval between neighboring pairs of time steps as the imaging device captures interferometric images. During the entire time of displacement measurement, the tensile machine keeps applying the load to the specimen causing its continuous deformation. Hence, the resultant displacement map can be effectively interpreted as representing the velocity field (the displacement occurring in the short time interval). Thus, hereafter, the displacement map is referred to as the velocity map.

#### 2.1.1. Acoustoelasticity

The first acoustic method employed in the present study is known as acoustoelasticity with a contact transducer. Details of the principle of acoustic elasticity can be found elsewhere [13,14,15,16,17,18]. Appendix A of this paper outlines it as well. Here, its essence is described in the context of the present study.

The velocity of an acoustic wave in an elastic medium is determined by the elastic modulus and density of the medium. In the linear elastic regime, the elastic modulus is a constant. If the medium is under a stress and the stress is so high that the displacement enters the nonlinear regime of the constitutive relation, the elastic modulus is not a constant anymore. This nonlinearity in the elastic modulus *E* can be expressed as a function of strain *ϵ* as follows.

(1) $$E=E_{0}+C^{\left(3\right)}\epsilon +\frac{1}{2}C^{\left(4\right)}\epsilon^{2}+\cdots +\frac{1}{(n-2)!}C^{\left(n\right)}\epsilon^{n-2}+\cdots$$

where $E_{0}$ is the elastic constant and $C^{\left(n\right)}$ is the $n^{th}$ order coefficient of the strain energy. Under this condition, the acoustic velocity can be expressed as follows.

(2) $$v^{ac}=\sqrt{\frac{E_{0}+C^{\left(3\right)}\epsilon }{\rho }}$$

Here in Equation (2), we consider up to the third order of the higher order terms in Equation (1). ($C^{\left(3\right)}$ is called the third order elastic constant, TOEC.) When a residual stress causes the nonlinearity, the relative acoustic velocity can be expressed with the residual strain $\epsilon_{res}$ and the TOEC as follows.

(3) $$v_{rel}^{ac}=\frac{v_{res}^{ac}}{v_{0}^{ac}}=\sqrt{\frac{E_{0}+C^{\left(3\right)}\epsilon_{res}}{E_{0}}}$$

where $v_{res}^{ac}$ and $v_{0}^{ac}$ are the acoustic velocity in a residually stressed specimen and a unstressed specimen of the same material. By measuring these velocities respectively with a contact-transducer and knowing the value of the TOEC, we can evaluate the residual strain by solving Equation (3) for $\epsilon_{res}$ as

(4) $$\epsilon_{res}=\frac{2E_{0}}{C^{\left(3\right)}}\left(v_{rel}^{ac}-1\right)$$

Once $\epsilon_{res}$ is found, we can evaluate the corresponding residual stress using the nonlinear elastic modulus Equation (1). See Appendix for more details regarding the derivation of these expressions.

**Figure 1:**
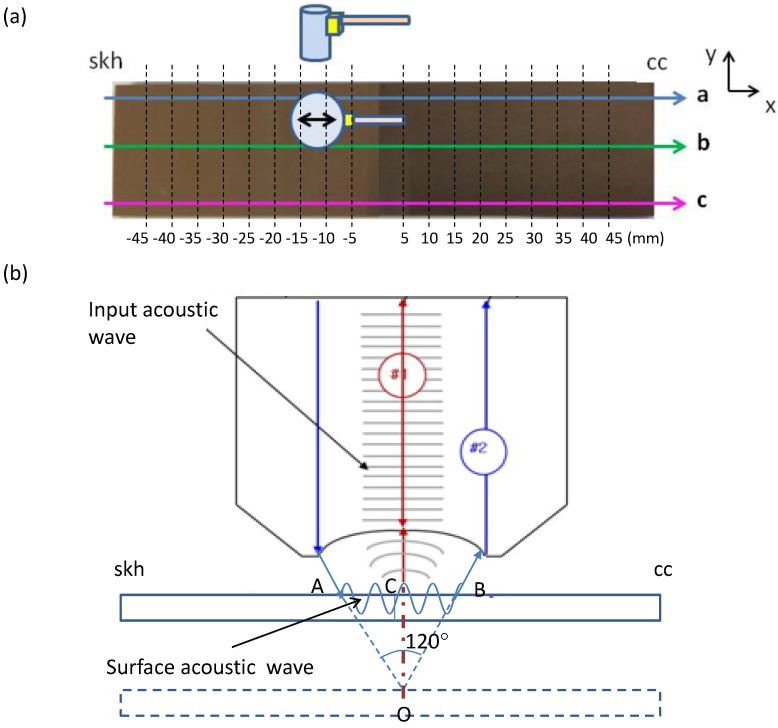
Acoustic sources used in this study. (**a**) Acoustic transducer. The sensor head was switched between the longitudinal-wave and shear-wave modes for the respective measurements. The grid points indicated on the specimen are the points where the acoustic measurements were made; (**b**) Point-focused acoustic source for Scanning Acoustic Microscopy. Acoustic frequencies of 400 and 200 MHz were used with the same focusing acoustic lens.

The second acoustic method is known as the V(z) analysis with Scanning Acoustic Microscopy (SAM). Details of its operation principle can be found in a number of references [24,25,26,27,28,29]. In short, it can be explained as follows. Refer to Figure 1b where a typical SAM setup is illustrated. The upper part of the drawing illustrates an acoustic focusing lens along with wavefronts of the input acoustic wave incident to the specimen placed below the lens. An acoustic transducer (not shown in the drawing) is placed on the top surface of the lens to send out the acoustic wave and receive the signal reflected off the specimen. When an acoustic wave is incident to the specimen surface through a layer of liquid (normally water) at an angle greater than the critical angle, the so-called leaky surface acoustic wave is generated and propagates along the liquid-solid interface.

Consider the two acoustic paths labeled #1 and #2 in Figure 1b. The former represents the acoustic wave that reaches the specimen surface and is specularly reflected off the surface back to the acoustic transducer through the acoustic lens. The latter represents the wave that propagates as the surface wave along the liquid-solid interface. The surface wave generates oscillatory motion at the liquid-solid interface, which radiates energy back into the liquid. The wave due to this acoustic radiation interferes with the specularly reflected wave at the top of the lens. The acoustic transducer detects the overall (superposed) wave and outputs as a voltage signal. Now consider that the specimen surface initially placed at the focal point of the acoustic beam is being displaced toward the lens. As the specimen gets closer to the lens, the specular reflection path #1 is shortened by 2OC. Here *O* is the focal point and *C* is the point where a vertical line originating from point *O* toward the lens crosses the top surface of the specimen. Path #2, on the other hand, loses the distance by AO+OB and gains by AB. As the distance between the specimen and the lens is varied, the two acoustic waves experience constructive and destructive interferences.

In other words, the voltage signal from the transducer *V* goes through crests (corresponding to constructive interference) and troughs (corresponding to destructive interference) as a function of *z*, the coordinate axis that measures the distance between the specimen and the lens. Thus, the voltage fluctuation associated with the interference is referred to as the V(z) curve. Since the frequency is fixed at the acoustic source, the acoustic path length (the path length in the unit of the wavelength) over AB depends on the phase velocity of the surface wave. The interval of these peaks (Δz) is associated with the velocity of the surface acoustic wave relative to the acoustic velocity in the coupling water as follows.

(5) $$V_{s}=\frac{V_{w}}{\sqrt{1-{\left(1-\frac{1}{2}\frac{V_{w}}{\Delta z}\cdot f\right)}^{2}}}$$

where $V_{s}$ is the surface acoustic wave velocity, $V_{w}$ is the acoustic velocity in water, and *f* is the acoustic frequency. The elastic modulus of the near surface region of the specimen can be characterized from $V_{s}$. This is how the elastic modulus is estimated with SAM. Some description regarding analysis of a V(z) curve is provided in the Appendix of this paper.

#### 2.1.2. Dynamic Analysis with Electronic Speckle-Pattern Interferometry (ESPI)

The optical method used in this study involves application of a low-level tensile load and strain measurement with the use of an optical interferometric technique known as the Electronic Speckle-Pattern Interferometry. This method makes use of the fact that in harmonic oscillation the acceleration is centripetal and hypothesizes that an elastic material exhibits harmonic oscillatory behavior even if a tensile load is applied at a rate much lower than the natural frequency of the harmonic system (This hypothesis is justified from the fact routinely observed in similar experiment that the fringe system keeps changing even after the tensile machine stops applying the load. In other words, the specimen behaves like a spring).

**Figure 2:**
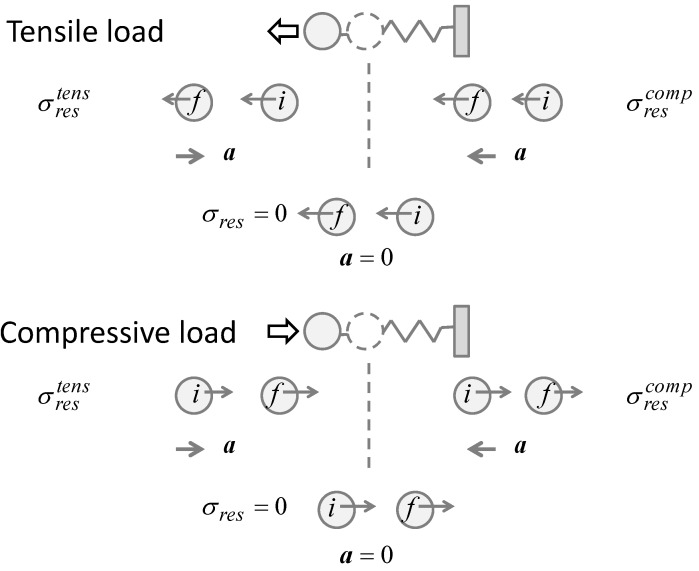
Spring-mass model to represent residual stresses and external forces.

Consider the dynamics of elasticity in Figure 2 that illustrates a simple spring-mass model. A given part of an elastic material can be modeled as a mass connected to the neighboring part of the material with a spring. If the part of interest is experiencing a compressive residual stress, the position of the mass is on the shorter inter-atomic distance side of the equilibrium position. Here, the equilibrium position corresponds to the situation where the spring is in the natural length and does not exert restoring force. Similarly, if the part is experiencing a tensile residual stress, its position is on the longer inter-atomic distance side of the equilibrium. Since these situations are caused by the residual stresses, these positions can be viewed as the initial position when an external force, a tensile force as an example, is applied. Upon the application of an external force, the residually-stressed part of the material behaves differently depending on whether the residual stress is tensile or compressive.

Using the spring-mass model illustrated in Figure 2, we can argue the behaviors as follows. The upper half of Figure 2 illustrates the situation where a tensile external force pulls the mass leftward. The dashed circle represents the mass at the equilibrium position. The masses labeled “i” and “f” respectively indicate the masses at the initial and final positions. The left picture illustrates the situation where the mass initially at a stretched position, representing a tensile residual stress, is further stretched by the external force and displaced to the final position. In this case, the external force pulls the mass further away from the equilibrium position, causing the spring to exert greater force in the opposite direction to the external force. Under the condition where the external force pulls the mass at a level just high enough to overcome the locking mechanism (A residual stress is locked under the equilibrium between the spring force and the counter force. If an external force (the third force) acts against the counter force, the equilibrium breaks and the mass is displaced in the direction of the external force), the leftward velocity of the mass decreases as it is displaced; the mass slows down as moving from the initial to final position. On the other hand, if the residual stress is compressive as illustrated by the right picture, the same external force displaces the mass toward the equilibrium point. This time, the external force is in the same direction as the spring force and the mass speeds up to the left.

This observation illustrates that when a tensile external force is applied, those regions with a tensile residual stress decrease the velocity and those with a compressive residual stress increase the velocity. The lower half of Figure 2 illustrates the situation where a compressive external force is applied to the mass. Repeating the same argument as above, we find that the mass speeds up if the spring is initially stretched and it slows down if the spring is initially compressed. Table 1 summarizes all the four combinations of the types of the residual stress and external force. The acceleration *a* indicates the change in the velocity for each case. If the acceleration is in the same direction as the external force, the mass speeds up; otherwise, it slows down. In terms of the direction, whether the external load is compressive or tensile, the resultant acceleration is leftward if the region has a compressive residual stress, and the acceleration is rightward if the region has a tensile residual stress. These patterns come from the fact that in a harmonic oscillation, the acceleration has always an opposite sign to the displacement from equilibrium. In other words, if the type of the external load (tensile or compressive) is the same as that of the residual stress, the material resists further by exerting acceleration in the opposite direction to the external load. If the types are opposite, the acceleration is the same in direction as the external force. This just represents the nature of centripetal force.

**Table 1:** Velocity change for different combinations of residual stresses and external loads.

External Force	*σ*_res_: Tensile			*σ*_res_: Compressive		
$F_{\leftarrow }^{tensile}$	$\overset{V^{0.2}}{\leftarrow }$	$\overset{V^{0.4}}{\leftarrow }$	$\overset{a}{\to }$	$\overset{V^{0.2}}{\leftarrow }$	$\overset{V^{0.4}}{\leftarrow }$	$\overset{a}{\leftarrow }$
$F_{\;\to }^{compressive}$	$\overset{V^{0.2}}{\to }$	$\overset{V^{0.4}}{\to }$	$\overset{a}{\to }$	$\overset{V^{0.2}}{\to }$	$\overset{V^{0.4}}{\to }$	$\overset{a}{\leftarrow }$

The above argument indicates that by monitoring the direction of the acceleration in association with the direction of the external load, it is possible to determine if a given part of the material has a residual stress and whether the residual is compressive or tensile. If the direction (sign) of the acceleration is the same as the tensile external load, the residual stress is compressive and if the direction of the acceleration is opposite to the tensile load the residual stress is tensile. With the use of ESPI, the acceleration can be evaluated as a two-dimensional full-field map via differentiation of the differential displacement (dξ) map with respect to time. Note that as mentioned above, the ESPI used in this study uses a small interval dt to evaluate dξ. Thus, the differential displacement map can be interpreted as representing the velocity dξ/dt.

### 2.2. Experimental Procedures

#### 2.2.1. Specimen Preparation

**Figure 3:**
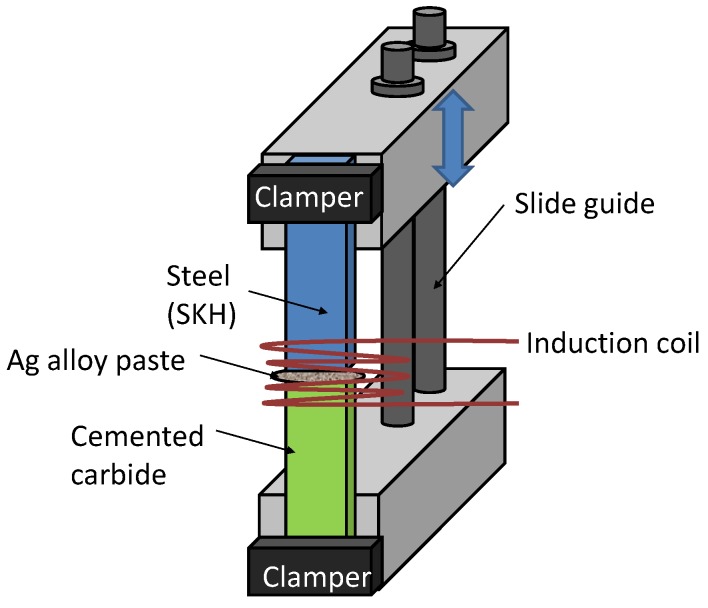
Butt-brazed specimen used in the present study.

A butt-brazed plate specimen shown in Figure 3 was used in the present study. The specimen was prepared in the following fashion. A high-speed tool steel (Japan Industrial Standard skh51, or ISO HS6-5-2 [33]. Called skh51 hereafter in this paper) plate of 18.5 mm wide, 50 mm long and 3.37 mm thick and a cemented carbide (cc) plate of the same dimension were placed on a mount with their 18.5 mm sides contacting with each other. Ag braze alloy paste (Ag-Cu-Zn-Ni alloy, ISO Ag450) was put at the contacting surface. An induction coil was set over a 30 mm-long region of the two plates centered at the 18.5 mm contacting sides. A braze temperature of 800 °C was kept for 10 s, followed by air cooling. The other 18.5 mm ends of the plates were clamped to blocks as shown in Figure 3. The two blocks were connected with slide guides where the upper block clamping the steel side of the specimen was able to slide freely during the brazing operation. Thus, the only constrain imposed to the plates during the brazing operation was gravity. After the brazing, the specimen was finished with grinding work. Table 2 lists pertinent material constants of the steel and cemented carbide.

**Table 2:** Material constants. $E_{elas}$: Elastic Modulus; *α*: Thermal Expansion; *κ*: Thermal Conductivity; $\sigma_{yield}$: Yields Stress. * cc shows brittle fracture characteristic and the value listed below as the yield stress is actually the ultimate strength.

Material	E_elas_ (GPa)	*α* (10^-6^· K^-1^)	*κ* (Wm^-1^K^-1^)	*σ*_yield_ (GPa)
skh 51	219	11.9	23.0	2.38
cc	580	5.3	67.0	1.5 *

#### 2.2.2. Acoustic Measurement

The butt-brazed specimen was first examined with the acoustic devices. Figure 1 illustrates (a) the contact acoustic (ultrasonic) transducer and (b) the Scanning Acoustic Microscope (SAM) used in the present study. The contact acoustic transducer was driven by a square wave pulser/receiver (Olympus 5077PR, Olympus, Tokyo, Japan) with transducer sensor heads for the longitudinal-wave mode (Olympus M110-RM) and the shear-wave mode (Olympus V156-RM). The sensor head was cylindrical with the circular contact area of 62.04 mm^2^. Since the specimen was 3.37 mm thick and the sensor evaluated the acoustic velocity through time-of-flight measurement of an acoustic pulse reflected from the rear surface, the strain gauge volume was 209.1 mm^3^. The SAM system (Olympus UH3) used a point-focused acoustic source operating at acoustic frequency of 400 and 200 MHz with the same acoustic lens of 310 μm focal length. This configuration generated a conical volume of acoustic field toward the focal point with an opening angle of 120°.

As Δz in the V(z) curve was evaluated when the focal point was in a range of 250 to 25 μm inside the specimen from the surface, the cross-sectional area that the acoustic pulse intersected with the specimen surface was a circle ranging from 860 to 86 μm in diameter. The acoustic wave in the ultrasonic range has penetration depth of the order of the wavelength. In the present case, it was 7.5 and 8.4 μm at 400 MHz for skh and cc, respectively, and twice of them at 200 MHz. These numbers can be translated into the maximum strain gauge volumes as 4.4 × 10^-3^ mm^3^ (cc), 4.9 × 10^-3^ mm^3^ (skh) for 400 MHz, and 8.8 × 10^-3^ mm^3^ (cc), 9.8 × 10^-3^ mm^3^ (skh) for 200 MHz. The minimum strain gauge volumes were one-hundredth of the respective cases.

For the coupling medium between the acoustic source and the specimen surface, Glycerin paste (Pony Industry, Co. Ltd., Osaka, Japan, SHN-B25) was used for the contact acoustic transducer with the shear-wave mode sensor head, and distilled water was used for the contact ultrasonic transducer with the longitudinal-wave mode sensor head and for the SAM. The measurement was made at 18 points along each of the three reference lines parallel to the 100 mm side of the specimen as shown in Figure 1. The 18 points of measurement were located every 5 mm starting from 5 mm from the end of the skh side all the way to 5 mm from the end of the cc side except at the middle point that fell on the brazing line.

#### 2.2.3. Displacement Measurement with ESPI

After the acoustic measurement was completed, the specimen was mounted on a tensile machine and analysis was made with the application of a tensile load at a constant pulling rate as high as approximately 400 N. Since the cross-sectional area of the specimen was 3.37 × 18.5 = 62.3 mm^2^ the averaged stress at the maximum load was approximately 400/62.3 × 10^-6^ N/m^2^ = 6.4 (MPa), which was more than two orders of magnitude lower than the yield stress of skh or cc as shown in Table 2.

An ESPI setup sensitive to in-plane displacement components parallel to the tensile axis (*x*-axis) and perpendicular to it (*y*-axis) was arranged in front of the tensile machine so that the differential displacement was measured as the tensile load was applied. A semiconductor laser (wavelength at 660 nm) beam was split into two interferometric paths with a beam splitter. The two steering mirrors placed in the respective optical paths steered the beams so that they would overlap each other on the specimen surface. The beam expanders were used so that the two beams would cover the area of interest on the specimen.

A phase shifter, comprising a glass disk and a turning mechanism to change the incident angle of the incoming beam to the optical wedge, was placed after one of the steering mirrors for the introduction of an artificial phase $\phi_{a}$. When switched from the base position to the turned position with the turning mechanism, the optical wedge introduced the artificial phase of $\phi_{a}\left(x\right)=ax$ on the specimen surface. Here *x* was the variable associated with the coordinate axis set parallel to the tensile axis, and *a* was a constant. The differential displacement measurement was conducted through the following steps.

Step 1: Using a digital camera, take an interferometric image of the specimen with the phase shifter at the base position and the two interferometric beams on top of each other on the specimen surface. Store the image data in computer memory (called the base image). Use this procedure for the first pair of the interferometric beams sensitive to the in-plane displacement component parallel to the tensile axis or the *x*-component (the *x* interferometer). Repeat the procedure for the second pair sensitive to the in-plane displacement perpendicular to the tensile axis and parallel to the braze line or the *y*-component (the *y* interferometer).
Step 2: Similarly to Step 1, take another interferometric image with the phase shifter at the turned position. Store the resultant image in computer memory (called the turned image). Take this procedure for the *x* interferometer first and repeat it for the *y* interferometer.
Step 3: Engage the tensile machine to apply a tensile load to the specimen. When the applied load reaches a preset value, take an interferometric image with the phase shifter at the turned position. Store the resultant image (called the deformed image) in computer memory. Use this procedure for the *x* and *y* interferometers respectively. Repeat the same procedure of taking a pair of *x* and *y* interferometric images at *N* more preset loads (at least one more load). While taking the pairs of images at preset loads, keep running the tensile machine with the same cross-head speed.

Step 1 through 3 are to acquire the interferometric image data. These steps yield two sets of images, one for the *x*-component and the other for the *y*-component of the in-plane displacement. Each set consists of a base image, a turned image and *N* deformed images. The following steps, the data analysis procedures, are taken after the data acquisition procedures have been completed. The data analysis procedures are common to both *x* and *y* components of the in-plane displacement. The displacement component below is denoted by *i*.

**Figure 4:**
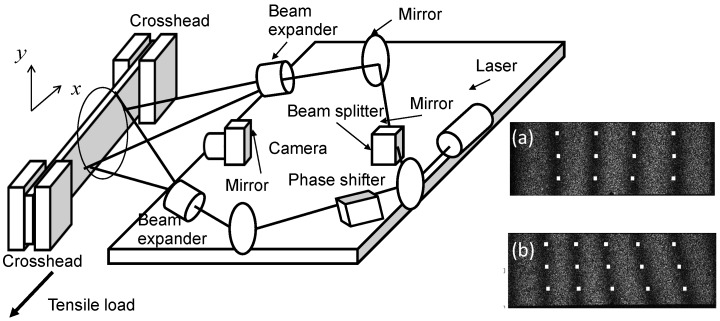
Optical arrangement for the electronic speckle-pattern interferometry (ESPI) setup. The illustration is for the *x* interferometer sensitive to the in-plane displacement component parallel to the tensile axis. An identical configuration for the *x*-component of the in-plane displacement called the *y* interferometer was configured. The *y* interferometer is not illustrated in this figure to avoid complexity. (**a**) Sample carrier fringe pattern; (**b**) Sample overall fringe pattern.

Step 4: Subtract the base image from the turned image on a pixel-by-pixel basis. Save the resultant subtracted image in computer memory. This subtracted image consists of mutually parallel equidistant dark fringes (stripes) referred to as the carrier fringe pattern. Figure 4a shows a sample carrier fringe pattern for the *x* interferometer. The fringes are equidistant and parallel because the artificial phase $\phi_{a}\left(x\right)$ is a linear function of *x* and independent of *y*.
Step 5: Subtract the base image from the deformed image on a pixel-by-pixel basis. Save the resultant subtracted image in computer memory. This subtracted image contains the phase information associated with both the artificial phase $\phi_{a}$ and the phase change due to the deformation caused by the tensile load, $\phi_{d}$. The image is called the overall fringe pattern. Figure 4b shows a sample overall fringe pattern for the *x* interferometer. Use this procedure *N* times, for each deformed image taken at the corresponding preset load.
Step 6: Assign an integer representing the fringe order to each of the fringes of the carrier fringe pattern and the overall fringe patterns, respectively (Here the fringe order is defined as follows. In accordance with the electronic speckle interferometry, each dark fringe represents the contour of a constant displacement that corresponds to the phase difference of 2nπ where *n* is an integer. Depending on the angle of incidence of the interferometric beams and other geometric factors, 2π corresponds to the unit displacement $\delta \xi_{i}$ where *ξ* denotes the displacement vector and $x_{i}$ is the coordinate variable that the pair of the interferometric beams is sensitive to. Assign n=0 to the dark fringe corresponding to zero displacement. Subsequently, assign n=1 or n=-1 to the fringe next to the fringe corresponding to n=0. Here if the next fringe corresponds displacement of $\delta \xi_{x}$, assign n=1 and if it corresponds to $-\delta \xi_{x}$, assign n=-1. Similarly, assign a signed integer to the next dark fringe one by one as 2nπ corresponds to $N\delta \xi_{i}$ (Figure 4).) Once all the fringes are assigned with an order, draw grid lines parallel to the *x*-axis at $y=y_{1}$ through $y=y_{k}$. For each grid line, find the coordinate points where each fringe crosses the grid line. This yields a vector $\left(x_{n},n\right)$ where $x_{n}$ is the *x* coordinate at which the grid line crosses the $n^{th}$ fringe. After all the grid lines are processed, generate a table containing the coordinate points where grid lines cross all the fringes. Here the $\left(x_{n},n\right)$ vector for the grid line at $y=y_{k}$ constitutes the $k^{th}$ row of the table. The resultant table is called the fringe order table. Generate a fringe order table for the respective components of the in-plane displacement vector, $\xi_{x}$ and $\xi_{y}$.
Step 7: Once a fringe order table is generated, replace the fringe order with corresponding displacement vector component according to the relation $\xi_{i}\left(n\right)=n\delta \xi_{i}$. Subsequently, for each row interpolate the vector $\left(x_{n},n\delta \xi_{i}\right)$ with respect to the discrete values of $x_{n}$ from the lowest to highest values of *n* for the row. Here, $\delta \xi_{i}$ is the unit displacement corresponding to one fringe interval (the displacement that causes the optical phase change of 2π). Iteration of this procedure for all the rows yields a map of $\xi_{i}$ that provides the displacement component $\xi_{i}$ for all the grid points.

On the image plane of the digital camera, the speckle size was comparable to or smaller than the pixel size. Since ESPI evaluated displacement using the phase change of each speckle, the strain gauge area was defined by the pixel size. On the object plane, each pixel covered an approximate area of 100 μm × 100 μm. To find the location of a dark fringe, approximately 10 pixels were involved perpendicular to the fringe (5 pixels for each side of the fringe). From all this information, the strain gauge volume can be estimated as 100 μm (parallel to the dark fringe) × 1000 μm (perpendicular to the dark fringe) × 3.37 mm (thickness) = 0.34 mm^3^. From the typical fringe interval (the distance between a pair of neighboring dark fringes) of 100 pixels and the involvement of 10 pixels perpendicular to each dark fringe (5 pixels per interval), the measurement error of the ESPI method can be estimated as 5%.

## 3. Results and Discussion

### 3.1. Acoustic Measurement

**Figure 5:**
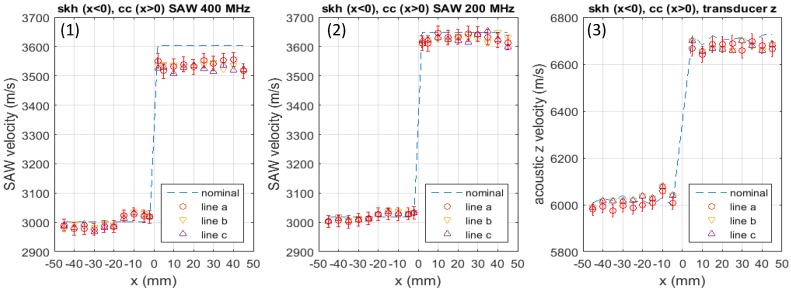
Acoustic velocity in *z* direction measured with (1) SAM 400 MHz, (2) SAM 200 MHz and (3) contact acoustic transducer.

Figure 5 plots the acoustic velocity measured at all reference points on the three lines a–c indicated in Figure 1. The three sets of graphs present measurement with (1) the Scanning Acoustic Microscope (SAM) operated at 400 MHz; (2) SAM operated at 200 MHz and (3) the contact acoustic-transducer operated in the longitudinal-wave mode, respectively. Since the dominant component of the surface acoustic waves generated in SAM is longitudinal, comparison of the SAM data with the contact transducer data is reasonable. Dashed lines present the nominal acoustic velocity measured with the respective methods for a non-brazed specimen of the same material and dimension as the brazed specimen. The butt-joint line is at x=0 mm, the skh side occurs for x<0 and the cc side occurs for x>0.

**Figure B1:**
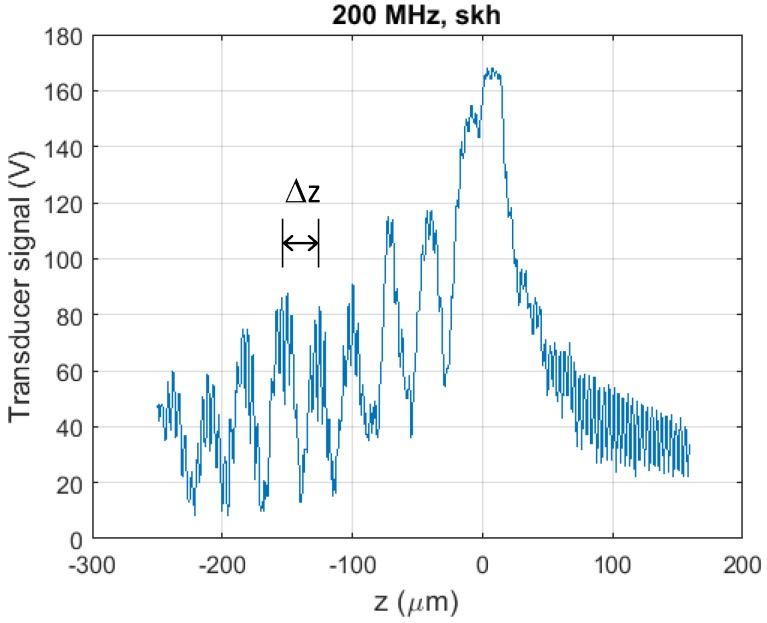
Typical V(z) curve. Surface acoustic-wave velocity can be obtained from Δz.

For each set of plots, the measurement error is indicated for the data along line a. Error bars are omitted for lines b and c for clarity, as the errors along these lines are the same as line a. Here, the error was estimated for each method as follows. In the present study, the dominant source of error in SAM was the finite step-width of the lens motion (the *z*-step) in association with the evaluation of Δz. The SAM instrument recorded the voltage signal at every $1/512^{th}$ of the entire distance that the acoustic lens moved for the generation of a V(z) curve. When Δz was evaluated from the interval between neighboring peaks of the V(z) curve (the interference peaks), the finite width of the *z*-step introduced uncertainty. In the case of 200 MHz SAM for skh (see Figure B1 in Appendix B ), for example, the interval between a neighboring pair of interference peaks was 28.5 μm whereas the *z*-step was 0.8 μm. This led to uncertainty of 0.8/28.5 = 2.8% for each pair of peaks.

In the actual analytical process, the V(z) curve was first Fourier transformed for removal of high frequency noise, and then Δz was evaluated via inverse Fourier-transform of the resultant frequency spectrum. This process involved all the interference peaks, reducing the uncertainty by the square root of the number of the peaks involved. In the 200 MHz SAM example, the involvement of 7 peaks reduced the uncertainty to $2.8/\sqrt{7}=1.0\%$. According to Equation (5), half of the uncertainty in Δz is propagated to the acoustic velocity. Based on this logic, the measurement error for the respective methods was estimated as listed in Table 3. Also listed in this table is the standard deviation in the acoustic velocity measured at five different points of the non-brazed specimen (the nominal acoustic velocity). The standard deviation appears less or comparable to the measurement error, indicating that the measurement error associated with structural variation of the specimen has a smaller effect.

The dominant source of the error in the acoustic velocity measurement with the contact transducer was the readout error. The acoustic signal detected by the sensor head was read out with an oscilloscope at a trigger frequency of 200 MHz, or the sampling time of 1/200 MHz = 5 ns. Since the longitudinal acoustic velocity in skh and cc were 6000 and 6700 m/s, the round trip times of the acoustic pulse were 1.12 and 1.01 μs, respectively. The finite sampling time and these round trip times gave rise to uncertainty of 0.45% (skh) and 0.50% (cc) in determining the acoustic velocity. Variation in the specimen thickness could be another source of the error. The nominal velocities shown in Figure 5 were taken at various points on the specimen, and their variation can be used to assess this effect. The standard deviation of the nominal velocity data is 0.23% (skh) and 0.24% (cc). Similar to the SAM case, the standard deviation was less than the estimated measurement error. These values are listed in Table 3.

**Table 3:** Error estimation and standard deviation.

Method	Error		Standard Deviation	
	skh 51	cc	skh 51	cc
400 MHz SAM	0.7%	0.7%	0.34%	1.2%
200 MHz SAM	0.5%	0.5%	0.15%	0.4%
Contact transducer	0.45%	0.5%	0.23%	0.24%

In all three graphs presented in Figure 5, it is commonly observed that on the skh side, the acoustic velocity is higher than the nominal value in the near butt-jointed region approximately x=-20 mm to x=0 mm from the joint line, and it is similar to the nominal value over the rest of the specimen. On the cc side, the acoustic velocity is lower than the nominal value uniformly over the entire region of the specimen. The observed change is especially prominent in the SAW 400 MHz case, where the increase in the acoustic velocity on the skh side is as high as 1%, and the decrease on the cc side is approximately 2%. In either case, there is no significant difference between the data for any two of the three lines a, b or c.

**Figure A1:**
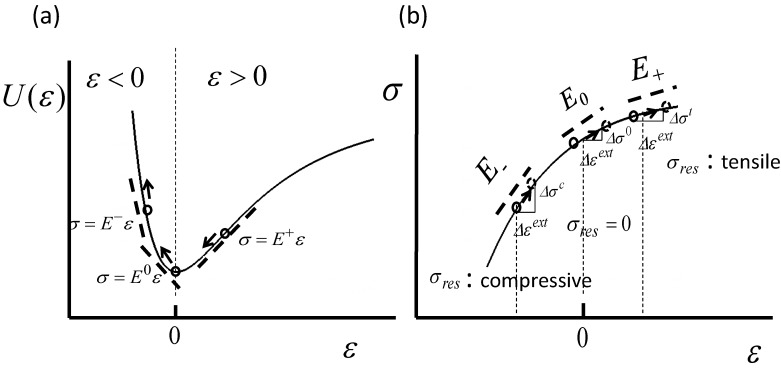
(**a**) Strain potential energy. The slope of the potential energy curve represents the stress; (**b**) The stress as a function of strain. The slope is the elastic modulus.

In accordance with Equation (3) and the expected change in the elastic modulus due to residual stresses discussed in Appendix A (See Figure A1), these observations can be interpreted as resulting from compressive residual stresses formed in the near butt-jointed region of the skh side and tensile residual stresses formed on the cc side. However, there is possibility that a change in the microstructure or some other effect induced by the brazing caused the observed change in the acoustic velocity. In the near-joint region on the skh side, in particular, it is likely that martensite was formed by the thermal load and the subsequent relatively fast cooling process. It is known that martensitic transformation changes the elastic property of the material and thereby affects the acoustic velocity.

To discuss these other effects, additional sets of experiment were conducted. Optical microscopic examination strongly indicated that the heating caused the formation of martensite. The Vickers hardness test showed about 50% increase in the hardness due to the heating, supporting the hypothesis of formation of martensite. It is known that martensite has a lower elastic modulus and density than ferrite. Since the reduction in the elastic constant is substantially greater than the reduction in the density, the acoustic velocity in martensite is lower than ferrite. Graye *et al.* [34] have reported about 0.7% reduction in acoustic velocity near the martensite quenched end of their specimen. The acoustic velocity measurements on the non-brazed specimen indicated that the heating caused 2% decrease in the skh and 1.4% decrease in the cc specimen. From this and the above observation that the acoustic velocity increased by 1% on the skh side and decreased by 2% on the cc side, we can say that the effect seen in Figure 5 is due to the residual stresses.

**Figure 6:**
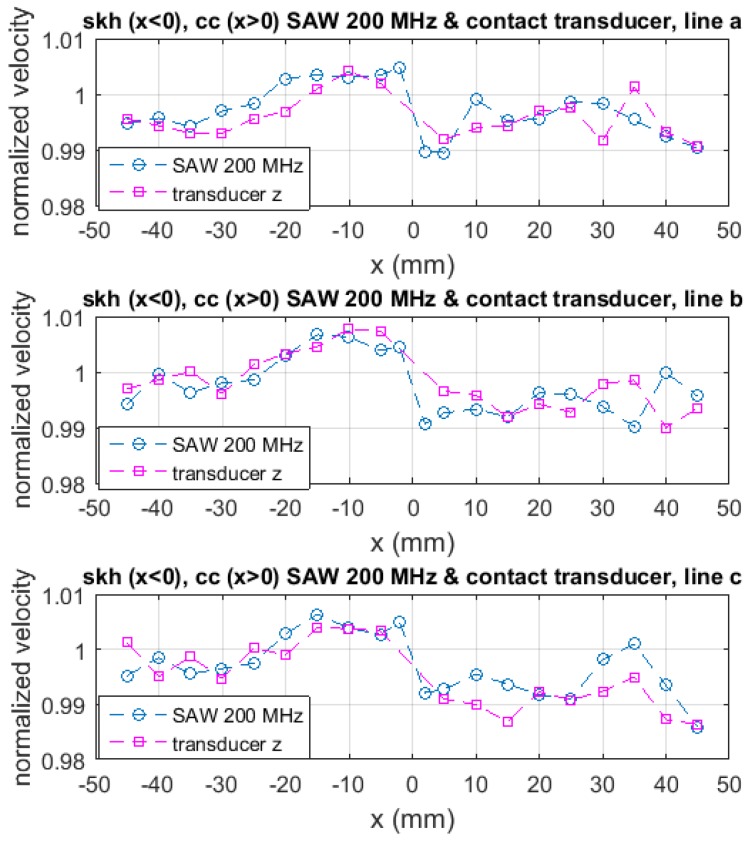
Relative acoustic velocity in *z* direction based on the measurement with the SAM 200 MHz and the contact acoustic transducer.

Comparison of Figure 5a through Figure 5c provides us with some information regarding the location of the residual stresses along the thickness. The 400 MHz SAM and 200 MHz SAM data were taken with the use of the same acoustic lens. Therefore, the angle of incidence to the specimen surface was the same. Under this condition, the penetration depth of the acoustic wave is inversely proportional to the acoustic frequency, being of the order of the acoustic wave length, *i.e.*, 15 μm for skh and 17 μm for cc. The longitudinal bulk acoustic-wave generated by the contact transducer passes through the specimen and reflects off the rear-surface. Thus, the acoustic velocity data represents the elastic modulus averaged over the entire thickness. The data with 400 MHz SAM shows much greater increase/decrease in the acoustic velocity from the nominal value than the 200 MHz SAM or the contact transducer. On the other hand, the data with the 200 MHz SAM and the contact transducer are similar to each other. Figure 6 explicitly shows this similarity in the form of $v_{rel}^{ac}$, the acoustic velocity normalized to the nominal value. These observations indicate that the residual stresses are concentrated within the region of 10 to 20 μm from the surface. The concentration of the residual stress near the surface is understandable because the induction heat by the brazing coil was loaded from the surface and the subsequent cooling was due to the natural dissipation from the surface. The post-brazing griding process is also a possible source of residual stresses near the surface.

**Figure 7:**
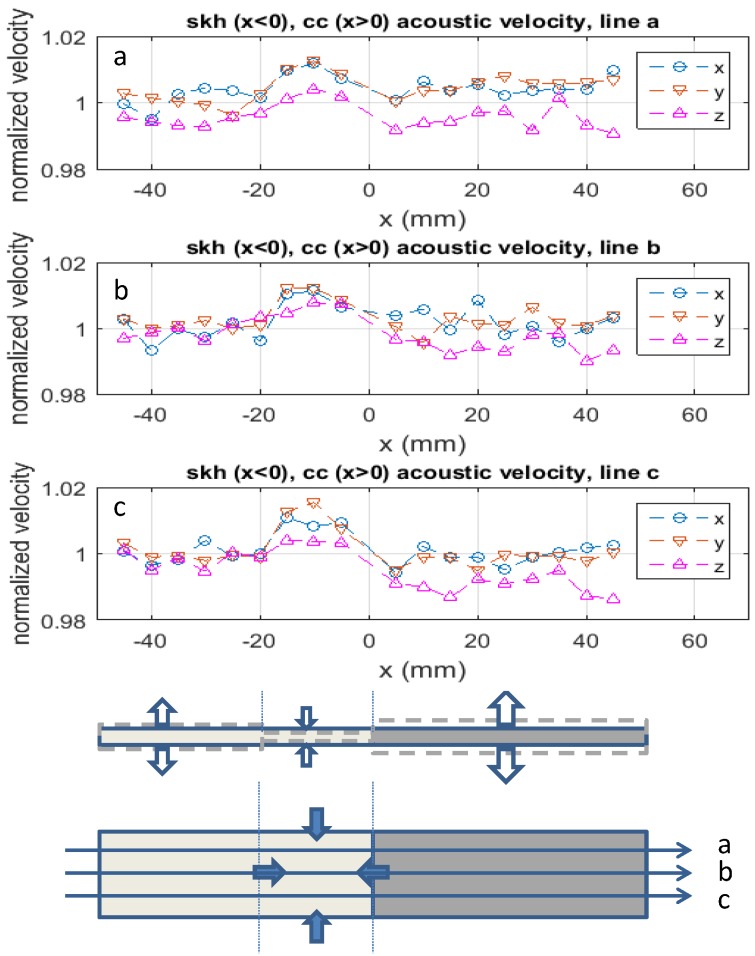
Relative acoustic velocity to nominal values for *x*, *y* and *z* direction (the top three rows). The bottom drawings illustrate compressive and tensile residual stresses qualitatively based on the relative acoustic velocity data.

Figure 7 plots the relative acoustic velocity measured with the contact acoustic transducer along the *x*-axis (parallel to the longer side of the specimen), *y*-axis (parallel to the butt-joint line) and *z*-axis (perpendicular to the xy-plane). The acoustic velocity profile along the *x* and *y* axes are similar to each other, indicating that during the heating and cooling processes, the specimen behaved symmetrically along these two axes. On the skh side, the relative acoustic velocity in the *x* and *y* direction is substantially higher than unity near the joint line (-20<x<0 mm) and approximately unity in the rest of the part toward the end of the specimen. On the cc side, on the other hand, they are slightly higher than unity along line a and approximately unity along lines b and c. These indicate that in the *x* and *y* directions the skh side was compressed in the near-brazed region and was barely compressed or stretched in the rest of the part, whereas on the cc side the specimen was slightly compressed along line a but barely affected along lines b and c. As for the *z* direction, the relative acoustic velocity is less than unity in most parts of the specimen except for -20<x<0 mm on the skh side where it is substantially higher than unity. This is the same region that the skh side shows the relative acoustic velocity higher than unity in the the *x* and *y* directions.

The drawing at the bottom of Figure 7 translates the observations in plots a, b and c in the relative acoustic velocity into compressive and tensile residual stresses qualitatively. The formation of the observed residual stresses can be explained as follows. As indicated in Table 2, the thermal conductivity of skh is a factor of three lower than cc. Therefore, when the induction coil heated up the specimen for brazing, the heat built up near the butt-joint line on the skh side. Consequently, this part of the skh material was thermally expanded and softened. The factor of two higher thermal expansion coefficient of skh than cc enhanced this localized thermal expansion. The gravity on the upper side of the specimen (Figure 3) compressed the softened part of the skh material. Due to the relatively low thermal conductivity, the other part of the skh side (toward the end of it) was not softened, and reacted to the gravitational force as a rigid body, confining the compression in the near brazed region. The factor of three lower elastic modulus of skh than cc enhanced this localized compression. This explains the compression in the *x* direction.

During this heating process, the near brazed region was thermally expanded in the *y* and *z* directions as well. During the cooling process, this region shrank as the temperature went down. Due to the lower thermal conductivity, the skh side shrank more slowly than the cc side. Consequently, after the shrinking process was over the near brazed region of skh was compressed by the cc side, which shrank more, and the rest of the skh side, which had not been thermally expanded. Again, the higher thermal expansion coefficient of skh enhanced this local compression effect. The relatively uniform profile of the relative acoustic velocity on the cc side can be explained by the higher thermal conductivity. When thermally loaded, this side of the specimen was heated relatively uniformly. The lower thermal expansion coefficient of cc limited the amount of thermal expansion during the heating process. During the cooling process, the higher thermal conductivity helped the heat dissipation on the cc side. Therefore, the cc side was almost instantaneously cooled off with the higher shrinking rate. Being smaller in dimension, this effect was higher along the thickness. This explains the tensile residual stress along the *z*-axis on the cc side; a tensile force was exerted along the thickness by the less shrinking skh side. The compressive residual stress along the *x* and *y* axes observed in the cc side can be ascribed to the Poisson’s effect associated with this enhanced tensile stress along the *z*-axis on the cc side.

### 3.2. Residual Strain and Stress Analysis

**Figure 8:**
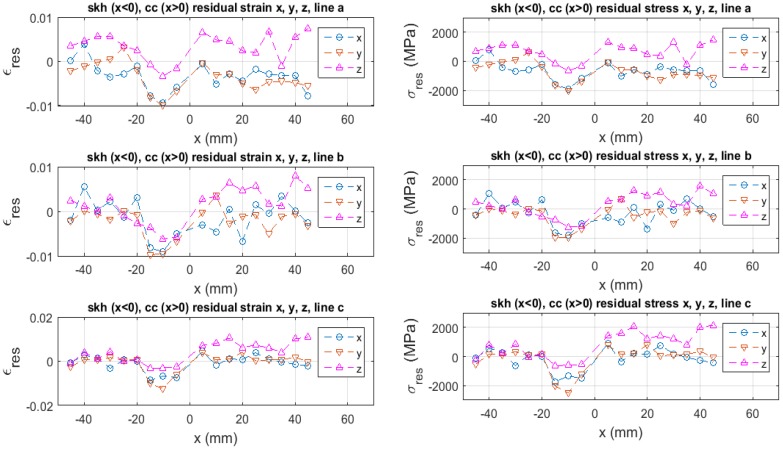
Residual strain based on relative acoustic velocity on Figure 7 and Equation (4), and corresponding residual stress evaluated with Equation (6).

Once the relative acoustic velocity is measured, we can estimate the residual strain with the use of Equation (4), provided that the elastic modulus $E_{0}$ and the third order elastic constant (TOEC) $C^{\left(3\right)}$ are known. Here, however, the TOEC $C^{\left(3\right)}$ is unknown for skh or cc, although the elastic modulus $E_{0}$ is known (Table 2). Some assumption is necessary for the use of Equation (4). The TOEF used by Muir [35] for low-carbon steel is a factor of two to three higher than the Young’s modulus (with a negative sign). Assuming that skh and cc have a similar strain potential function to low-carbon steel, we put $C^{\left(3\right)}=-2.5E_{0}$ in Equation (4) and estimate the residual strain. The left graph of Figure 8 plots the residual strain based on this assumption. Here, the tensile strain is positive and the compressive strain is negative. The top, middle and bottom rows are, respectively, the data obtained along line a–c. The maximum compressive residual strain of approximately 1.5% is seen in the *y* direction (parallel to the butt-joint line) along line c near the brazed region of the skh side (x=-10 mm). The tensile residual strain as high as 1% is seen on the cc side.

The right graph of Figure 8 is the corresponding residual stress evaluated as (See Equation (A7) in Appendix for the derivation of Equation (6)).

(6) $$\sigma_{res}=E_{0}\epsilon_{res}+\frac{1}{2}C^{\left(3\right)}\epsilon_{res}^{2}$$

The compressive residual stress as high as 2.5 GPa is seen in the *y* direction (parallel to the butt-joint line) along line c near the butt-joint line on the skh side (x=-10 mm). The tensile residual stress as high as 2.1 GPa is seen in the *z* direction (along the thickness) on the cc side. The observed maximum compressive residual stress is comparable to the yield stress of skh, 2.38 GPa. The observed maximum tensile residual stress is substantially higher than the ultimate strength of cc, 1.5 GPa (This value was measured in the present study).

A possible explanation of this tensile residual stress 50% or so higher than the ultimate stress is that the actual effect of the TOEC on the tensile side is higher than the assumed value ($2.5E_{0}$); in other words, the deviation of the strain potential curve from the quadratic dependence is higher on the tensile strain side. This speculation is plausible if we consider that when the residual stress is formed the material is already plastically deformed to some extent. Our previous study on a butt-welded steel specimen [21] indicates some evidence that the specimen is plastically deformed when it has residual stresses at a level lower than the present case.

**Figure 9:**
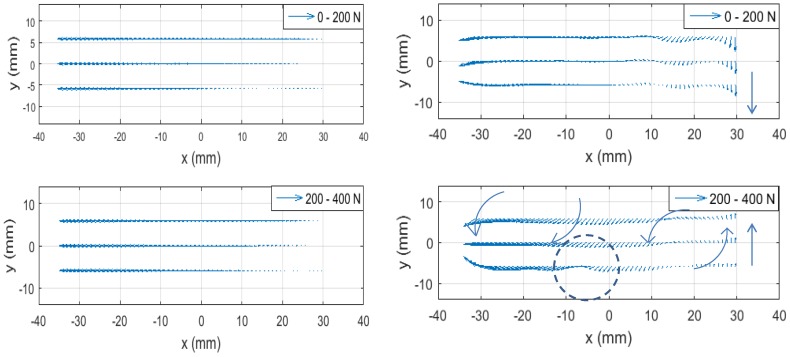
Velocity field 0–200 N (**top**) and 200–400 N (**bottom**). In the right figure, the vertical component of velocity is multiplied by a factor of 500 for better visibility of rotational feature.

The effect of the residual stress can be discussed in the context of the material’s response to the external force. The left graphs of Figure 9 show quiver plots of the displacement vector evaluated with ESPI for the tensile load increase from 0 to 200 N (top) and 200 to 400 N (bottom). As described above, these displacements data are taken while the tensile machine keeps pulling the specimen. So, the physical quantity represented by these plots is proportional to the velocity. The velocity component parallel to the tensile axis is much higher than the component perpendicular to the tensile axis. For better visualization of the material’s behavior perpendicular to the tensile axis, in the right two graphs the vertical component (the component perpendicular to the tensile axis) is multiplied by a factor of 500. Three features are worth paying attention to.

First, the velocity field of 200 to 400 N deformation shows rotation (vortex) like features. Normally, when a tensile load is applied, a plate specimen undergoes uniform deformation along the tensile axis and symmetric compression in the orthogonal direction reflecting Poisson’s effect. The observed rotation-like features can be explained as follows. Due to the altered elastic modulus, the specimen is unable to undergo symmetric compressive deformation in the orthogonal direction to the tensile axis. Our previous experimental study [21] on a butt-welded, thin-plate specimen similar to the present study indicates that the hindrance of natural compression perpendicular to the tensile axis generates stress concentration at a certain point away from the weld line, and the resultant weakness at the point causes bodily rotation of the specimen. It is likely that the present specimen shows a similar behavior.

Second, the velocity field of 200 to 400 N deformation shows a stagnant feature along line c (the bottom reference line) near x=-7 mm, as highlighted with a dashed circle in Figure 9. The cause of this feature is seen in Figure 8 in the region next to -9<x<-16 mm along line c where the compressive residual stress in the *y* direction (perpendicular to the tensile axis) is at the maximum; that is, the altered elastic modulus is the highest in the *y* direction. The stagnant behavior of the velocity field near x=-7 mm can be explained as follows. Because of the elevated elastic modulus, the specimen is unable to get compressed in the *y* direction as it would normally do when the tensile force is applied, and consequently, experiences counterclockwise bodily-rotation on the left side of the circled area with a hinge around x=-7 mm near the bottom side.

Third, near the right end of the specimen, the vertical velocity vector is seen to change the direction from downward (the top plots for the 0 to 200 N deformation) to upward (the bottom plots for the 200 to 400 N deformation). This indicates that this end of the specimen oscillates with the increase of the tensile load. Such an oscillatory motion is consistent with the transverse wave dynamics of plasticity as theoretically derived [23] and experimentally confirmed in a tensile experiment with a constant pulling rate similar to this study [36]. The swinging up motion of the right end during the 200 to 400 N deformation causes the clockwise bodily-rotation on the right-side of the hinge at x=-7 mm. Since the tensile machine does not exert torque, the specimen undergoes the counterclockwise rotation on other side of the hinge so that the total angular momentum remains null.

Finally, we can briefly discuss the above hypothesis regarding the detection of residual stress based on the sign of acceleration (see Table 1 and related arguments), using the experimental results. In Table 1, define the rightward direction as the positive direction. According to this table, whether the external load is tensile or compressive, a compressive residual stress generates negative acceleration and a tensile residual stress generates positive acceleration. According to the argument made in conjunction with the strain potential energy (Figure A1), on the other hand, a compressive residual stress increases the acoustic velocity and a tensile residual stress decreases it. From these two arguments, it follows that those regions where the acoustic velocity is higher than the nominal value should coincide with the region where the acceleration is negative, and the regions where the acoustic velocity is lower should coincide with positive acceleration.

**Figure 10:**
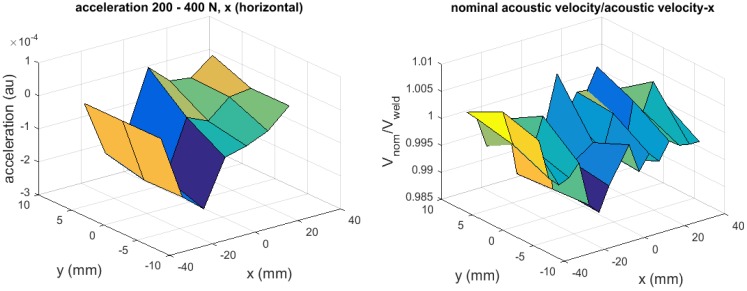
Acceleration from electronic speckle-pattern interferometry (ESPI) measurement and acoustic velocity relative to nominal value.

Figure 10 compares the acceleration (left graph) and the relative acoustic velocity (right graph) as three-dimensional plots. Here the acceleration data is evaluated by subtracting the velocity plots taken when the tensile load is increased from 0 to 200 N from the velocity plots taken when the tensile load is increased from 200 to 400 N. Note that in order to make the positive acceleration (the tensile residual stress) correspond to the lower relative acoustic velocity, the vertical axis of the right graph is in the unit of “nominal velocity/acoustic velocity” (so that the ratio is higher for the tensile residual stresses than the compressive residual stresses). The two graphs show qualitative similarity in the overall shape, demonstrating the consistence with the hypothesis.

## 4. Conclusions

Application of acoustoelasticity and optical interferometry to analysis of residual stresses has been discussed. For the demonstration of the methodology, a butt-brazed dissimilar plate specimen has been used. A Surface Acoustic Microscope (SAM) and longitudinal and shear contact acoustic wave transducers have been used for the acoustoelastic analysis and Electronic Speckle-Pattern Interferometry (ESPI) combined with monotonic tensile loading at a constant pulling rate has been used for the optical analysis. The acoustoelastic analysis has provided us with the information regarding the changes in the elastic modulus caused by residual stresses, and the ESPI analysis has allowed us to understand dynamic behavior of the specimen under the influence of the residual stresses. The use of the different acoustic sources has revealed that the residual stresses are concentrated within the subsurface region of the order of a few tens of microns from the surface. The spatial profile of the residual stresses has been quantitatively explained based on the mechanical and thermal properties of the dissimilar materials.

The ESPI analysis on the in-plane acceleration field supports our hypothesis that regions of compressive or tensile residual stresses can be revealed as two-dimensional full-field information. Although preliminary, this result encourages us to conduct further study on the idea of using the acceleration field to quantify residual stresses. At this point, the observed consistency between the acceleration and the relative acoustic velocity (Figure 10) supports the hypothesis qualitatively. An important area of future research in this regard is to extend the idea to quantitatively connect the acceleration with the relative acoustic velocity. One idea we have in mind is that we find the residual stress value absolutely at reference points via acoustoelasticity (with the precise knowledge of the third order elastic constant) and estimate the residual stress values at all points based on the acceleration of a given point relative to that of the reference points. In principle, this methodology is applicable to residual stresses in general.

## Appendix A. Acoustoelasticity

Consider the inter-atomic interaction referring to Figure A1a where elastic-strain potential-energy is illustrated as a function of strain *ϵ*. The point labeled ϵ=0 denotes the bottom of the potential well, which corresponds to the equilibrium point of the inter-atomic distance where the atom is most stable. On the right-hand side of the equilibrium, the atom is pulled away from the neighboring atom, or equivalently speaking, the material experiences a local tensile strain. Similarly, the left-hand side of the potential well corresponds to a local compressive strain. The three little circles shown on the potential curve illustrate representative positions on the shorter inter-atomic distance side of the equilibrium (left circle), near the equilibrium (middle circle), and on the the longer inter-atomic distance side of the equilibrium (right circle), at which the atom is positioned by a certain mechanism. The arrows illustrate the situation where a compressive force pushes the atom toward the shorter inter-atomic distance side. In the context of residual stress, the atom is positioned at the respective inter-atomic distances due to a compressive residual stress (left circle), no residual stress (middle circle) and a tensile residual stress (right circle), and the compressive force is due to an external load. As the atom is displaced by the external load to a new position on the potential curve, the local stress-strain condition changes accordingly.

The tangential lines shown near the circles in Figure A1a represent the slopes of the potential curve at the respective points. Mathematically, the slope represents the derivative of the potential energy with respect to the strain. Physically, the derivative represents the stress, whose dependence on the strain is determined by the constitutive relation as follows.

(A1) $$\frac{dU(\epsilon )}{d\epsilon }=\sigma \left(\epsilon \right)$$

If the potential function is quadratic, the constitutive relation Equation (A3) is a linear function of *ϵ*, *i.e.*, *E* is a constant, as shown below.

(A2) $$\begin{array}{ccc} U(\epsilon ) & = & \frac{E}{2}\epsilon^{2} \end{array}$$

(A3) $$\begin{array}{ccc} \sigma (\epsilon ) & = & \frac{d}{d\epsilon }\frac{E\epsilon^{2}}{2}=E\epsilon \end{array}$$

In most solid materials, the potential curve is approximately quadratic near the equilibrium, and therefore, they exhibit linear elasticity Equation (A3) when the strain is small. However, it is not unusual that a residual stress causes the deformation to surpass the linear range.

Figure A1b illustrates the situation explicitly where a residually-stressed material undergoes deformation due to an external load. If the residual stress is compressive, the initial inter-atomic position is to the left of the equilibrium. In this case the dependence of the potential curve on the strain is greater than quadratic, as seen in Figure A1, and hence the local elastic modulus is greater than that of the linear elastic regime near the equilibrium. On the contrary, if the residual stress is tensile, the local elastic modulus is less than the linear regime. The slopes labeled $E_{-}$, $E_{0}$ and $E_{+}$ indicate this situation that the elastic modulus depends on the type of residual stress. If an external load causes tensile strain, as the arrows in Figure A1b illustrates, the resulting stress-change depends on the residual stress. In Figure A1b, the stress-change caused by the same external-load-induced strain $\Delta \epsilon^{ext}$ is illustrated schematically for the compressive residual-stress case ($\Delta \sigma^{c}$), no residual-stress case ($\Delta \sigma^{0}$), and the tensile residual-stress case ($\Delta \sigma^{t}$). If the residual stress is compressive, the stress change is greater than the case of no residual stress. Adversely, the greater stress is necessary to cause the same strain as compared with the non-residual stress case. On the contrary, if the residual stress is tensile, the stress-change for a given strain is lower and the stress necessary to cause the same strain is lower as compared with the no residual-stress case. Once the external load is removed, the stress is relaxed as much as the stress-change due to the external load. The situation is the same if the external load causes a compressive strain.

Thus, it is possible to assess the residual stress by evaluating the local elastic modulus point-by-point on the specimen. Acoustoelastic techniques evaluate the local elastic modulus via acoustic velocity measurement based on the following relation of the acoustic velocity to the elastic modulus.

(A4) $$v^{ac}=\sqrt{\frac{E}{\rho }}$$

Here $v^{ac}$ is the phase velocity of the acoustic wave transmitted through the material, *E* is the elastic modulus, and *ρ* is the density of the material. In the context of Figure A1b, the external load in this case is the oscillatory force due to the acoustic wave. The elastic modulus *E* is associated with the type of the acoustic wave; if the wave is longitudinal *E* represents the Young’s modulus and if the wave is shear it represents the shear modulus. Figure 1a schematically illustrates the arrangement of the acoustic velocity measurement with the single transducer configuration in the shear wave mode with the acoustic oscillation along the *x*-axis. The transducer emits a pulsed acoustic signal on the front surface and receives the pulse after it is reflected off the rear surface. From the time-of-flight analysis, the acoustic velocity can be evaluated. By rotating the shear mode transducer by $90^{\circ }$, the acoustic velocity of the shear wave with the acoustic oscillation along the *y*-axis can be evaluated. Similarly, by replacing the shear mode transducer with a longitudinal mode transducer, the acoustic velocity of the longitudinal acoustic wave can be evaluated.

With the existence of a residual stress, the elastic modulus is different from the nominal value (the value with no residual stress) as discussed above, and the acoustic velocity is altered accordingly. The relative change in the acoustic velocity can be expressed, as follows.

(A5) $$v_{rel}^{ac}=\frac{v_{meas}^{ac}}{v_{0}^{ac}}=\frac{\sqrt{\frac{E_{res}}{\rho }}}{\sqrt{\frac{E_{0}}{\rho }}}=\sqrt{\frac{E_{res}}{E_{0}}}$$

Here, $v_{rel}^{ac}$ is the relative change in the acoustic velocity due to the residual stress, $v_{meas}^{ac}$ is the acoustic velocity measured in a residually-stressed specimen, $v_{0}^{ac}$ is the nominal acoustic velocity, $E_{res}$ is the elastic modulus altered by the residual stress from the nominal value $E_{0}$. For quantitative evaluation of residual stress, the function U(ϵ) must be precisely known. Normally, U(ϵ) is Taylor-expanded with respect to strain *ϵ* and approximated with the terms as high as the third-order as follows.

(A6) $$U=C^{\left(0\right)}+C^{\left(1\right)}\epsilon +\frac{1}{2}C^{\left(2\right)}\epsilon^{2}+\frac{1}{6}C^{\left(3\right)}\epsilon^{3}$$

Accordingly, the stress and the elastic modulus can be expressed as follows.

(A7) $$\begin{array}{ccc} \sigma & = & \frac{dU}{d\epsilon }=C^{\left(1\right)}+C^{\left(2\right)}\epsilon +\frac{1}{2}C^{\left(3\right)}\epsilon^{2}=E_{0}\epsilon +\frac{1}{2}C^{\left(3\right)}\epsilon^{2} \end{array}$$

(A8) $$\begin{array}{ccc} E & = & \frac{d\sigma }{d\epsilon }=E_{0}+C^{\left(3\right)}\epsilon \end{array}$$

Here $C^{\left(1\right)}$ is the stress unrelated to strain, and $C^{\left(2\right)}$ is the nominal elastic modulus $E_{0}$. In the present context, the strain-unrelated stress is insignificant, and put to null in Equation (A7). Substitution of Equation (A8) into $E_{res}$ of Equation (A5) leads to

(A9) $$v_{rel}^{ac}=\sqrt{\frac{E_{0}+C^{\left(3\right)}\epsilon_{res}}{E_{0}}}={\left(1+\frac{C^{\left(3\right)}\epsilon_{res}}{E_{0}}\right)}^{1/2}\approx 1+\frac{C^{\left(3\right)}}{2E_{0}}\epsilon_{res}$$

where $\epsilon_{res}$ is the residual strain that alters the elastic modulus from the nominal value $E_{0}$ to $E_{res}$. From the measured value of $v_{rel}^{ac}$ and a known value of $C^{\left(3\right)}$ (called the third order elastic constant, TOEC), we can evaluate the residual strain by solving Equation (A9) for $\epsilon_{res}$.

(A10) $$\epsilon_{res}\approx \frac{2E_{0}}{C^{\left(3\right)}}\left(v_{rel}^{ac}-1\right)$$

Subsequently, the corresponding residual stress can be evaluated with the use of Equation (6).

## Appendix B. V(z) Curve

Figure B1 is a typical V(z curve obtained by SAM. The horizontal axis (*z*) represents the distance between the acoustic lens and the specimen as the lens position is moved relative to the specimen surface. The highest peak at z=0 μm corresponds to the situation where the acoustic wave is focused on the specimen surface. The other peaks correspond to the lens positions that generate constructive interference between the specularly reflected acoustic wave and the surface-radiated acoustic wave.

As seen in Figure B1, high frequency noise is often superposed to a V(z) curve, and it affects the accuracy in the evaluation of Δz. Fourier transform is widely used to remove those high frequency components in the frequency domain. After the noise removal, Δz is evaluated by inversely Fourier transforming the frequency domain data.
